# 1518. Prevalence of Integrase Inhibitor Resistance within an Urban Clinic Network After Adoption as First-line Therapy

**DOI:** 10.1093/ofid/ofad500.1353

**Published:** 2023-11-27

**Authors:** Jenna E Januszka, Emily N Drweiga, Melissa E Badowski

**Affiliations:** University of Illinois Chicago College of Pharmacy, DURHAM, North Carolina; University of Illinois at Chicago College of Pharmacy, Chicago, Illinois; University of Illinois Chicago, Chicago, Illinois

## Abstract

**Background:**

Limited data outside of randomized controlled trials exist reporting the prevalence of integrase inhibitor resistance (INSTI-R) since the approval and recommendation of INSTIs as first-line treatment for HIV. National surveillance data in 2018 estimated INSTI-R to be 6.3% in people with HIV (PWH) who had never achieved viral suppression, with 0.8% attributed to transmitted drug resistance (TDR). The purpose of this study was to describe the prevalence of INSTI-R in patients from a single urban clinic network on INSTI-containing regimens. Prevalence amongst University of Illinois Chicago Community Clinic Network (UCCN) patients on INSTI-containing single-tablet regimens specifically was previously reported.

**Methods:**

This was a retrospective study of adult PWH followed at UCCN prescribed an INSTI-containing regimen between September 2017 and September 2020 with > two recorded HIV-1 RNA viral loads collected > 12 months apart. The primary endpoint was the difference in INSTI-R in UCCN patients compared to national data. Other outcomes included development of virologic failure (VF), defined as > 200 copies/mL in two consecutive HIV-1 RNA viral loads, and patient specific factors associated with VF. The primary endpoint was analyzed using a chi-square test. All other data are presented as descriptive statistics.

**Results:**

Of 948 patients screened, 321 were included and followed an average of 30 months. Baseline characteristics are presented in **Table 1.** A total of 5 subjects had INSTI-R prior to switch resulting in a prevalence of 1.6%, which was significantly less than the national prevalence of 6.3% (p< 0.001). Of note, all patients with INSTI-R were previously treated with first generation INSTIs and none were deemed TDR. Study-defined VF occurred in 26 subjects (8.1%). Seven subjects (26.9%) were not taking antiretrovirals for > six weeks at the time of study-defined VF. Outcomes of patients with VF are outlined in **Table 2.** Subjects with a pre-treatment viral load > 100,000 were more likely to experience VF (p=0.0485).

**Figure:**
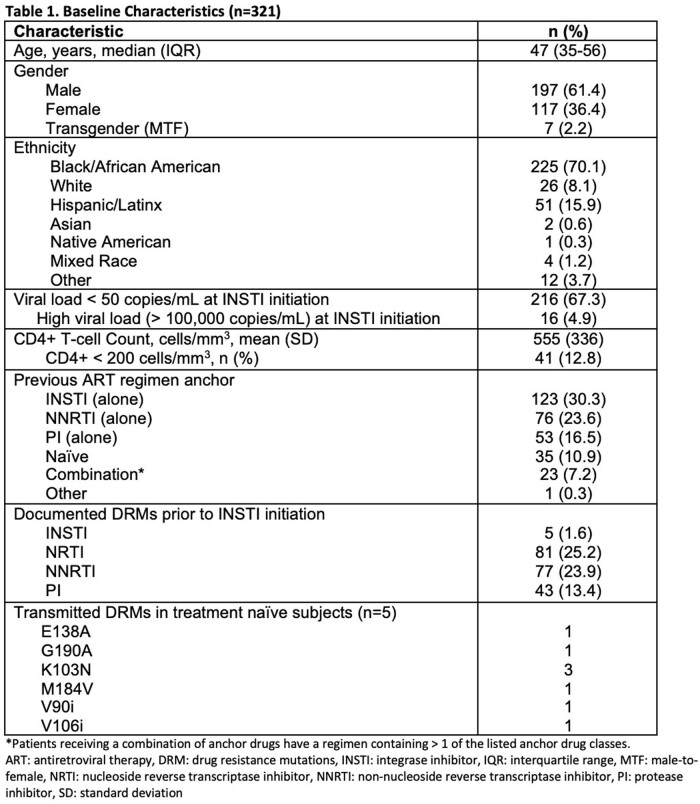

**Figure:**
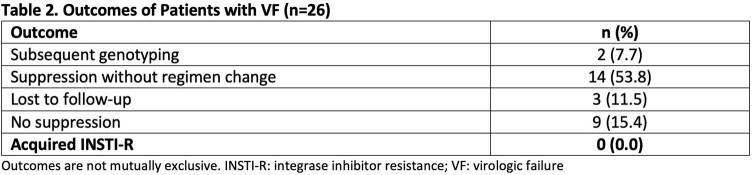

**Conclusion:**

Among UCCN patients on INSTI-containing regimens, INSTI-R rates were lower than the estimated national prevalence; however, this comparison is limited due to the large proportion of subjects with viral suppression at baseline.

**Disclosures:**

**All Authors**: No reported disclosures

